# Tumor Characteristics and Event Free Survival in Older and Younger Women with Early Breast Cancer

**DOI:** 10.3390/curroncol33090503

**Published:** 2026-08-25

**Authors:** Samantha Kodikara, Alexis C. Wardell, Allison M. Deal, Annie Page, Hyman B. Muss, Kirsten A. Nyrop

**Affiliations:** 1 School of Medicine, University of North Carolina at Chapel Hill, Chapel Hill, NC 27599, USA; samkodikara@gmail.com (S.K.); hyman_muss@med.unc.edu (H.B.M.); 2 Lineberger Comprehensive Cancer Center, University of North Carolina at Chapel Hill, Chapel Hill, NC 27599, USA; ally_wardell@med.unc.edu (A.C.W.); allison_deal@med.unc.edu (A.M.D.); 3 Gillings School of Global Public Health, University of North Carolina at Chapel Hill, Chapel Hill, NC 27599, USA; apage@rti.org

**Keywords:** tumor characteristics, event free survival, breast cancer, older and younger women

## Abstract

**Background:** Age, race, body mass index (BMI), breast density, parity, smoking and alcohol use are associated with increased risk for breast cancer. These factors, as well as tumor characteristics, treatment regimens and comorbidities, are analyzed for associations with five-year event free survival (EFS) in a sample of women with Stage I-III breast cancer who received chemotherapy with curative intent. **Methods**: EFS was defined in terms of breast cancer recurrence, second primary, metastasis, and overall survival. Analyses were stratified by age (under age 65 vs. over age 65). EFS was estimated using the Kaplan–Meier method and compared using a Cox proportional hazard model. **Results**: In a sample of 821 women, mean age at diagnosis was 54 years, with 75% White and 22% Black. Younger women had higher proportions of Stage II and III tumors (*p* = 0.005), larger tumor size (*p* = 0.0004), and higher breast density (*p* = 0.003). Five-year EFS was 91% among younger vs. 82% among older women (*p* = 0.0005). In women aged < 65, there were 48 EFS events, and triple negative patients had significantly worse EFS compared to other subtypes (*p* = 0.003). Smokers also had worse EFS (*p* = 0.04). In women aged ≥ 65, there were 26 events, and both tumor size (*p* = 0.02) and mastectomy (*p* = 0.03) were significant for EFS. **Conclusions**: In our sample, triple negative subtype, smoking history, tumor size, and surgery type were significantly associated with shorter EFS. Race, BMI, alcohol use, parity, breast density, radiation treatment, and specific chemotherapy regimen were not significant for EFS in either age group.

## Simple Summary

Age, race, body mass index (BMI), breast density, parity, smoking and alcohol use are associated with increased risk for breast cancer. These factors, as well as tumor characteristics, treatment regimens and comorbidities, are analyzed with regard to associations with five-year event free survival (EFS) in a sample of women with Stage I-III breast cancer who received chemotherapy with curative intent and compared by age group (under age 65 vs. age 65 and older). EFS was defined in terms of breast cancer recurrence, second primary, metastasis, and overall survival. Triple negative subtype, smoking history, tumor size, and surgery type were significantly associated with shorter EFS. Race, BMI, health behaviors, parity, breast density, and specific chemotherapy regimen were not significant for EFS in either age group.

## 1. Introduction

Breast cancer is largely a disease of aging, with 67 the median age at diagnosis [1]. Breast cancer subtypes are generally less serious with advancing age, but older women can nevertheless have higher risk tumors that can impact their prognosis and survival [2].

There are many risk factors for female breast cancer beyond advancing age [3]. High mammographic breast density (MBD), observed in 34% of U.S. women age 40–74 [4], is a risk factor [5] and is largely hereditary [6]; however, 20–30% of the variance in MBD is explained by age, Body Mass Index (BMI) and/or parity [7]. MBD generally declines with advancing age [8]. The association of MBD with BMI is similarly inverse, with high MBD observed among 59% of women with normal BMI and 25% among women with obesity [4]. Higher parity is inversely associated with MBD in both pre- and postmenopausal women [9].

Independent of its association with breast density, obesity (BMI of 30 or higher) as compared to normal weight (BMI < 25) is associated with an increased risk for breast cancer, especially in women who are postmenopausal [10,11]. High BMI is also associated with increased risk for Stage III/IV breast cancer and Grade 3/4 tumors [12], and in Black women, it is associated with an increased risk for triple negative tumors [13]. High proportions of women are overweight or have obesity at breast cancer diagnosis [14], and it is common for women to gain weight after they have completed primary treatment [15,16,17,18]. Therefore, it is an added concern when high breast density is coupled with unhealthy high BMI [19,20,21,22,23]. Heterogeneous or extreme breast density can be an increased risk for invasive breast cancer if the BMI is 25 or higher [21], especially for postmenopausal women [24]. The combination of overweight/obese body composition and elevated breast density in premenopausal women is similarly associated with a higher risk of ER-negative breast cancer vs. ER-positive breast cancer [25].

This study assessed how a variety of factors that increase the risk for breast cancer diagnosis can also be associated with event free survival (EFS) within 5 years post-diagnosis. This was investigated in a sample of women with Stage I-III breast cancer who received chemotherapy with curative intent. Our specific aim was to investigate how these factors impact prognosis and survival in older (age 65 or older) as compared to younger women (under age 65). EFS was defined in terms of breast cancer recurrence, second primary, metastasis, and survival. EFS was evaluated for associations with age, race, BMI, breast density, parity, smoking and alcohol use as well as tumor characteristics, treatment regimens and comorbidities.

## 2. Materials and Methods

Study Participants. The data set for this study was initially created to evaluate weight trajectories in women receiving treatment for early breast cancer at a single institution, the North Carolina Cancer Center in Chapel Hill (IRB 15-1523). The study subjects were identified from a consecutive review of clinic appointments, with 8% diagnosed 1992–2005 and 92% diagnosed 2006–2016. Further details regarding the sample were published previously [1,2,3]. For the current analysis, the sample was limited to women who received chemotherapy with curative intent and for whom complete data were available within five years of breast cancer diagnosis (not “lost to follow-up”).

Measures. The timeline was pre-treatment to either a cancer-related “event” or censure at five years (no further event monitoring after five years), whichever was earlier. Cancer-related events were defined as breast cancer recurrence, second primary, metastasis, and survival.

Electronic Medical Record. Electronic Medical Records (EMR) data uploaded into a REDCap© database included age, race, BMI, comorbidities, parity, history of smoking, and alcohol use. Data pertaining to breast cancer diagnosis included stage, oncotype, subtype, tumor size, and breast density score. Chemotherapy treatment was documented by regimen and receipt of radiation as yes/no.

Statistical Considerations. Demographic and clinical characteristics were compared across age groups (younger < 65 vs. older ≥ 65). Fisher’s exact test was used for categorical variables and Kruskal–Wallis for continuous variables. Patients were stratified by age for analysis (under age 65 versus 65 or older), and outcomes were censored at 5 years post-diagnosis. EFS was estimated using the Kaplan–Meier method and compared using log-rank tests and univariable and multivariable Cox proportional hazards models. Cox proportional hazard models were adjusted for diagnosis stage and tumor size. Kaplan–Meier plots and estimates, as well as hazard ratios (HR) along with 95% confidence intervals are reported. All analyses were conducted in SAS version 9.4 (SAS Institute, Cary, NC, USA).

## 3. Results

### 3.1. Sample Characteristics (Table 1)

In the sample of 821 women, the mean age at diagnosis was 54 years (range 23–83); 79% were under age 65, and 21% were age 65 or older at diagnosis. Seventy-five percent identified as White, 22% as Black, and 3% as Other. Twenty-four percent of the under 65 cohort identified as Black as compared to 17% of the 65 or older cohort (*p* = 0.02).

Health and Health Behavior. The mean BMI at diagnosis was 29 (range 18–59). The mean number of comorbidities for women under age 65 was 0.8 (range 0–6), and for women age 65 or older, it was 2.2 (range 0–6) (*p* < 0.0001). The mean number of obesity-related comorbidities was also higher among younger women (1.3 vs. 0.5) (*p* < 0.0001). Women under 65 had an 82% parity rate as compared to 91% of women aged 65 or older (*p* = 0.004). Thirty-six percent of women < 65 had a past or current history of smoking compared to 47% of women 65 or older (*p* = 0.002). The history of alcohol use did not differ between age groups.

Breast Cancer Diagnosis. The breast cancer stage among women < 65 was I (20%), II (51%) or III (29%) as compared to patients aged 65 or older with stage I (32%), II (47%), or III (22%) (*p* = 0.005). Younger women had a comparatively larger tumor size (3.3 vs. 2.8) (*p* = 0.0004) and higher breast density (*p* = 0.003). There were no significant inter-group differences in breast cancer subtype.

Breast Cancer Treatment. A higher proportion of younger as compared to older patients received a mastectomy (57% vs. 41%) (*p* = 0.0007). Eighty-five percent of patients received the four most common chemotherapy regimens: 48% AC-T (doxorubicin/cyclophosphamide followed or preceded by paclitaxel), 6% AC = TC (AC-T plus anti-HER2), 23% TC (docetaxel/cyclophosphamide plus or minus anti-HER2), and 10% TCH (docetaxel/carboplatin plus anti-HER2). Higher proportions of younger patients received anthracycline-based sequential regimens (AC-T, AC-TC) (*p* < 0.0001). There were no significant intergroup differences in the proportion receiving radiation treatment.

**Table 1:** Sample characteristics.

Variable	Overall N = 821	Under Age 65 N = 646	Age 65 and Older N = 175	*p* Value *
**Demographics**				
Age—mean, SD, range	54 (SD 11.9) 23–83	50 (SD 9.4) 23–64	70 (SD 4.0) 65–83	**<0.0001**
Race White Black Other	590 (74.8%) 173 (21.9%) 26 (3.3%)	446 (72.6%) 144 (23.5%) 24 (3.9%)	144 (82.3%) 29 (16.6%) 2 (1.1%)	**0.02**
**Health and Health Behavior**				
BMI at diagnosis—mean, SD, range	29 (6.7) 18–59	29 (6.9) 18–59	29 (5.7) 19–48	0.39
Total comorbidities (mean, SD, range)	1.1 (1.3) 0–6	0.8 (1.1) 0–6	2.2 (1.4) 0–6	**<0.0001**
Obesity-related comorbidities—(N, %)—mean, SD	0.7 (0.9) 0–4	0.5 (0.8) 0–4	1.3 (1.0) 0–4	**<0.0001**
Parity Yes No	625 (83.9%) 120 (16.1%)	478 (81.8%) 106 (18.2%)	147 (91.3%) 14 (8.7%)	**0.004**
History of smoking Yes No	306 (38.4%) 491 (61.6%)	228 (36.2%) 402 (63.8%)	78 (46.7%) 89 (53.3%)	**0.02**
History of alcohol use Yes No	317 (43.5%) 412 (56.5%)	243 (42.3%) 331 (57.7%)	74 (47.7%) 81 (52.3%)	0.24
**Breast Cancer Diagnosis**				
Breast cancer stage I II III	186 (22.7%) 408 (49.7%) 226 (27.6%)	131 (20.3%) 327 (50.6%) 188 (29.1%)	55 (31.6%) 81 (46.6%) 38 (21.8%)	**0.005**
Tumor size (mean, SD, range), centimeters	3.2 (SD 2.7) 0.1–26.0	3.3 (SD 2.6) 0.1–20.0	2.8 (SD 2.9) 0.2–26.0	**0.0004**
Mammographic Breast Density (MD) score A B C D	30 (4.3%) 315 (45.4%) 299 (43.0%) 51 (7.3%)	20 (3.7%) 236 (43.5%) 238 (43.9%) 48 (8.9%)	10 (6.5%) 79 (51.6%) 61 (39.9%) 3 (2.0%)	**0.003**
Breast cancer subtype HR−/HER2− HR−/HER2+ HR+/HER2− HR+/Her2+	188 (23.0%) 66 (8.1%) 415 (50.8%) 148 (18.1%)	145 (22.6%) 53 (8.2%) 331 (51.5%) 114 (17.7%)	43 (24.7%) 13 (7.5%) 84 (48.3%) 34 (19.5%)	0.82
**Breast Cancer Treatment**				
Surgery Mastectomy Lumpectomy Neither	424 (51.9%) 390 (47.7%) 3 (0.4%)	356 (55.3%) 286 (44.4%) 2 (0.3%)	68 (39.3%) 104 (60.1%) 1 (0.6%)	**0.0007**
Chemotherapy regimen—drug combinations AC-T (doxorubicin/cyclophosphamide followed/preceded by paclitaxel/Taxol) AC-TC + anti-HER-2 therapy TC (docetaxel/cyclophosphamide) +/− anti-HER-2 therapy TCH (docetaxel/carboplatin + anti-HER-2 therapy) Other	395 (48.4%) 46 (5.6%) 185 (22.7%) 78 (9.6%) 112 (13.7%)	347 (54.0%) 40 (6.2%) 118 (18.4%) 59 (9.2%) 78 (12.2%)	48 (27.6%) 6 (3.4%) 67 (38.5%) 19 (10.9%) 34 (19.6%)	**<0.0001**
Radiation Yes No	658 (80.8%) 156 (19.2%)	517 (80.7%) 124 (19.3%)	141 (81.5%) 32 (18.5%)	0.91

* Fisher’s exact for categorical variables and Kruskal–Wallis for continuous. Bold print highlights statistical significance.

### 3.2. Five-Year EFS and OS Stratified by Age

Since the EFS curves were significantly different between patients under age 65 versus 65 and above (*p* = 0.0005), all Table 1 variables were analyzed for associations with EFS separately for each age group.

**Figure 1:**
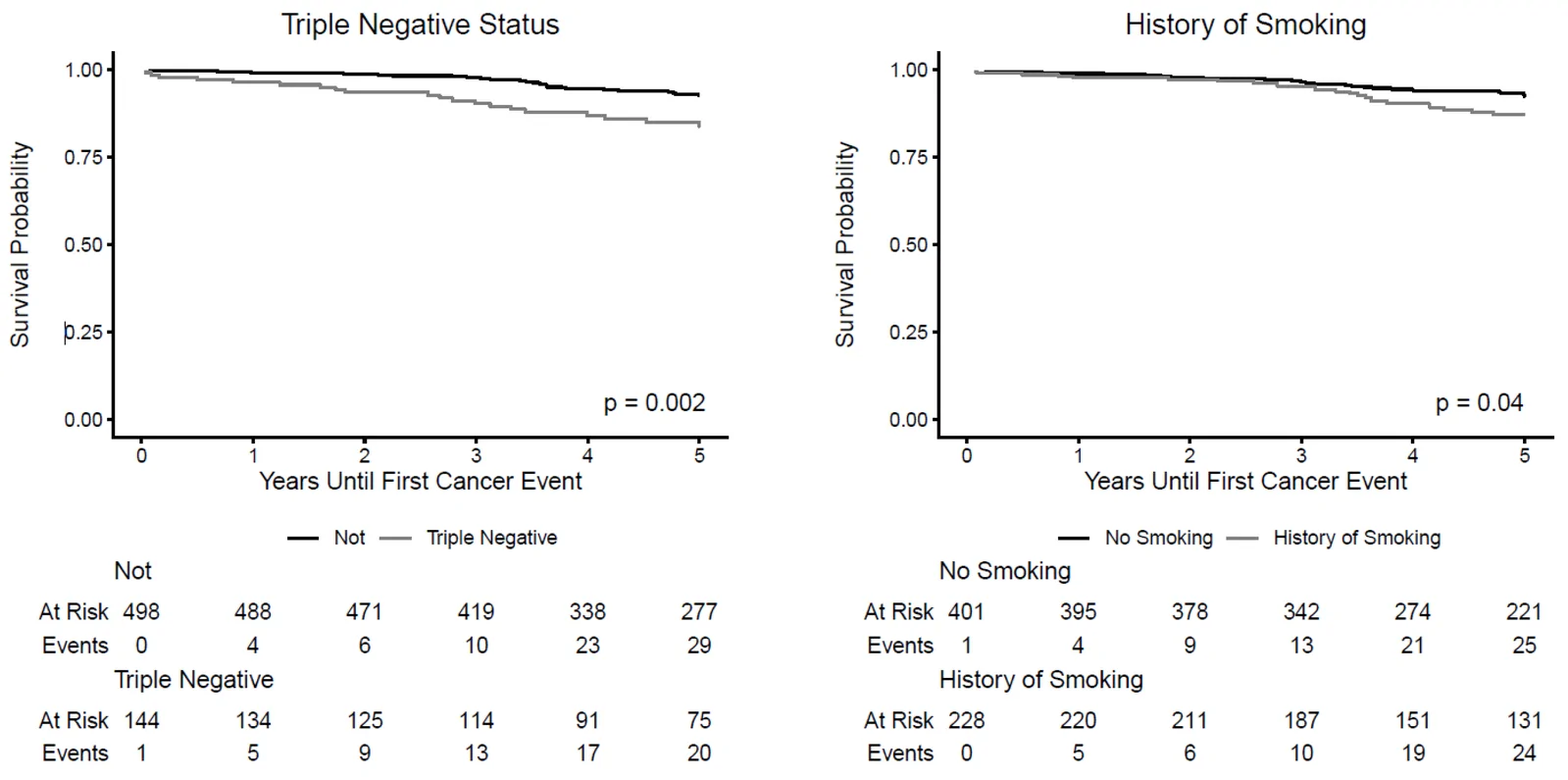
Event free survival—women under age 65.

Among patients under age 65, there were 49 events. Of all variables analyzed for associations with EFS in this age group, only smoking history (HR: 1.8, 95% CI 1.0–3.2, *p* = 0.04) and triple negative subtype were significant compared to those other subtypes (HR: 2.4, 95% CI 1.3–4.3, *p* = 0.01) (Figure 1). After adjusting for diagnosis stage and tumor size, the triple negative subtype remained significant (HR: 2.2, 95% CI 1.2–4.0, *p* = 0.01). The five-year EFS rate was 93% (89–95%) with no smoking history, compared to 87% (82–91%) with a smoking history. The five-year EFS rate was 93% (90–95%) for other than triple negative subtype, compared to 84% (77–90%) for triple negative. Of all variables analyzed for associations with overall survival (OS) in this age group, only the triple negative subtype was significant compared to other subtypes (HR: 5.4, 95% CI 2.2–13.1, *p* = 0.0002).

**Figure 2:**
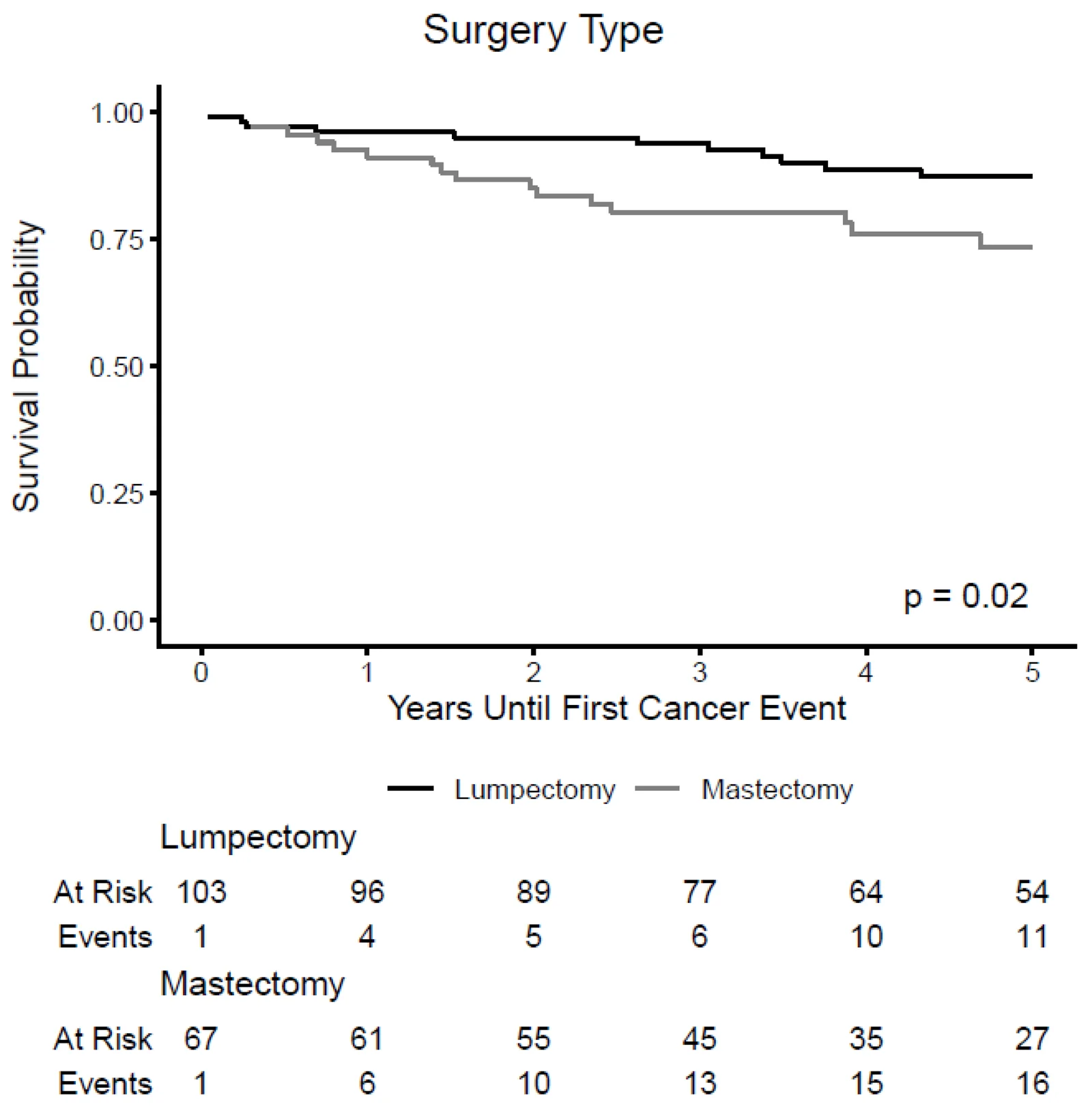
Event free survival—women age 65 or older.

Among patients aged 65 and older, there were 27 events, and there was a 10% increase in hazard for every cm change in tumor size (HR: 1.1, 95% CI 1.0–1.2, *p* = 0.02). The surgery type was significant for EFS; patients who received a mastectomy had a worse EFS compared to patients who received a lumpectomy (HR: 2.5, 95% CI 1.1–5.5, *p* = 0.03). The five-year EFS was 75% (61–84%) for patients who received a mastectomy compared to 88% (79–94%) for patients who received a lumpectomy. Of all variables analyzed for associations with OS in this age group, only the triple negative subtype was significant compared to other subtypes (HR: 4.2, 95% CI 1.5–12.0, *p* = 0.008) (Figure 2). After adjusting for the diagnosis stage and tumor size, the triple negative subtype became significant for EFS (HR: 2.9, 95% CI 1.2–6.9, *p* = 0.01).

Within both age groups, there were no significant associations with EFS and OS by race, BMI, alcohol use, parity, breast density, radiation treatment, or chemotherapy regimen.

## 4. Discussion

In this study, we evaluated the five-year EFS in women with early breast cancer who received chemotherapy with curative intent. Our research question was whether factors known to contribute to an initial breast cancer diagnosis were also significant factors for prognosis and survival. We also investigated tumor characteristics and treatments for associations with EFS. Our specific interest was a comparison of older (age 65 or older) and younger women (under age 65).

In our sample, younger women had significantly fewer total and obesity-related comorbidities, lower parity, a lower percent with smoking history, a higher breast cancer stage, a larger tumor size, and higher breast density. Younger women also had higher proportions of mastectomies and anthracycline-based sequential regimens.

Among women under age 65, the variables significantly associated with a lower EFS were a smoking history and triple negative breast cancer. For women aged 65 and older, the tumor size at diagnosis and mastectomy were significantly associated with a lower EFS. Race, BMI, alcohol use, parity, breast density, and specific chemotherapy regimen were not significant for EFS in either age group.

Triple negative breast cancer (TNBC) is a tumor subtype that is especially susceptible to recurrence or disease progression in all age groups [24]. It is also more frequently diagnosed in younger as compared to older women [25,26]. In our study, the TNBC sample was relatively small within the older age group. After adjusting the TNBC analysis for stage and tumor size (which differed significantly between age groups), both younger (HR: 2.2; 95% CI 1.2–4.0; *p* = 0.01) and older (HR: 2.9; 95% CI 1.2–6.9; *p* = 0.01) women had a poorer five-year EFS.

The link between smoking history and cancers of the lung, trachea, bronchus, month, throat, stomach and esophagus is well established [27], while evidence of a direct or indirect (via substandard diet and/or excess inactivity) link between smoking and breast cancer is more modest [28,29]. However, the evidence of a link is stronger in women who started smoking at an early age, especially before their first pregnancy [30]. The concern is that exposure of breast tissue to potent carcinogens at an early age can contribute to the development of more aggressive treatment-resistant cancers—such as TNBC—characterized by rapid cell proliferation. Our finding that both TNBC and smoking history were significant for a lower EFS supports the association.

In the cohort of older women, the association of larger tumors size and mastectomy with lower EFS suggests a subgroup where this surgery choice was the best option in light of more advanced disease stage at diagnosis and/or overall health and frailty concerns. For any age group, a larger tumor size is associated with decreased disease-free survival [31]. Studies have also reported worse survival in older women with a larger tumor size, regardless of surgery choice [32] or when adjusted for aggressive tumor features [33].

In our study, we did not find an association of high BMI with EFS in either age group. The evidence to date is mixed regarding associations of obesity or adiposity with breast cancer prognosis and survival [34,35]. Specifically, the evidence is considered moderate in premenopausal women [36]; there are some inconsistencies in observed associations of BMI at diagnosis and survival by race and ethnicity [37,38,39], and there is potentially an “obesity paradox” of improved survival among women with higher BMI who have advanced breast cancer [40]. Other studies have reported a substantially higher risk of dying in women with obesity at diagnosis as compared to women with normal weight [41], a higher risk of dying independent of breast cancer subtype [42], and a higher risk of breast cancer recurrence [43].

Prior studies have also shown mixed results with regard to alcohol consumption and prognosis and survival [44,45,46,47,48,49,50]. We did not find an association between parity and survival (our older cohort had significantly higher parity compared to younger women) as observed in some studies [51,52]. The association of breast density with prognosis and survival is still an evolving area of research [53,54].

The strength of our study is the wide range of factors we evaluated for associations with five-year EFS, although our sample may not have been large enough to analyze some of these factors. Additional limitations of our study include a single-institution sample and five-year timeframe, resulting in relatively few “events” within each age group. We were also not able to consider patients who were lost to phone or email follow-up and who may or may not be significantly different from our final sample. More definitive findings could be obtained from a larger multi-site sample and a longer follow-up period.

## Data Availability

The data presented in this study are available on request from the corresponding author.
